# Effectiveness of quadrivalent HPV vaccination in reducing vaccine-type and nonvaccine-type high risk HPV infection

**DOI:** 10.1017/S0950268823000213

**Published:** 2023-02-15

**Authors:** Chenxi Li, Thomas G. Hall, John J. Hall, Wen-Qiang He

**Affiliations:** 1 Melbourne School of Population & Global Health, The University of Melbourne, Melbourne, 3053, Australia; 2 Children's Hospital at Westmead, Westmead, 2145, Australia; 3 School of Population Health, UNSW Sydney, Sydney, 2052, Australia; 4 Childrens Hospital at Westmead Clinical School, The University of Sydney, Sydney, 2145, Australia

**Keywords:** cross protection, human papillomavirus, nonvaccine type high-risk HPV, quadrivalent vaccine, vaccine effectiveness, vaccine type HPV

## Abstract

This study aimed to assess human papillomavirus (HPV) vaccine effectiveness (VE) against both vaccine-type and nonvaccine-type high-risk HPV (hrHPV) infection, and duration of protection in United States. The study population was female participants aged 18–35 years with an HPV vaccination history and genital testing for HPV from the National Health and Nutrition Examination Survey, 2007–2016. Participants vaccinated before sexual debut were assessed against 13 nonvaccine-type hrHPV infection including 31/33/35/39/45/51/52/56/58/59/68/73/82. Multivariable logistic regression was used to estimate VE overall, by age at diagnosis, time since vaccination and lifetime sexual partners. A total of 3866 women were included in the analysis, with 23.3% (95% CI 21.3%–25.4%) having been vaccinated (≥1 dose). VE against vaccine-type HPV18/16/11/6 infection was 58% overall, which was mainly driven by those aged 18–22 years (VE = 64%) and 23–27 years (65%). Among participants aged 18–22 years vaccinated before sexual debut, the VE was 47% (23%–64%) against 13 nonvaccine-type hrHPV and 61% (95% CI 36%–77%) against 5 selected nonvaccine-type hrHPV35/39/52/58/59. Both direct effectiveness and cross-protection maintained effective for 5–10 years post vaccination. We also found the prevalence of ever diagnosed cervical cancer among vaccinated was significantly lower (0.46%, 4/874) than that among unvaccinated participants (1.27%, 38/2992). These findings highlight the potential of significant reduction of cervical cancer following the universal HPV vaccination programme.

## Introduction

Human papillomavirus (HPV) infection is the most common sexually transmitted infection in the United States (US) with estimated 42.5 million persons infected with some type of HPV and half of new infections occurring before age 24 years [1]. Although most infections resolve without clinical sequelae, persistent HPV infection can cause cervical cancer, other anogenital and oropharyngeal cancers. Based on their oncogenic potential, 15 different HPV types have been classified as high-risk HPV (hrHPV), including types 16/18/31/33/35/39/45/51/52/56/58/59/68/73/82 [2]. Of these, HPV16 and HPV18 are the most carcinogenic HPV types, causing about 70% of cervical cancer [3] and are targeted by all three licensed HPV vaccines, i.e., bivalent, quadrivalent and nonavalent [4]. And evidence has suggested that vaccination prior to the start of sexual activity is most effective [5].

Since 2006, HPV vaccine has been recommended in US for adolescent girls at age 11–12 years, with catch-up vaccination recommended through age 26 years [6]. Quadrivalent HPV vaccine was most commonly used through 2016 [7]. Following the universal HPV vaccination programme, the prevalence of vaccine-type HPV infection has decreased significantly over the past several years from 11.5% in 2003–2006 to 1.1% in 2015–2018 according to data from the National Health and Nutrition Examination Survey (NHANES) in US [8–11]. However, most of the studies only reported the prevalence of HPV infection and prevalence ratio (PR) by comparing to the prevalence in pre-vaccine era [8–10].

Based on real-world data from other countries, the vaccine effectiveness (VE) against vaccine-type HPV infection ranged between 73% and 100% among girls or younger women aged between 12 and 21 years [12–14]. To our knowledge, the duration of protection is still unclear although no waning immunity was found after 12 years of vaccination [15]. In addition, one study has investigated cross-protection against nonvaccine-type hrHPV infection among young women aged 20–26 years but found higher prevalence of nonvaccine-type hrHPV among vaccinated women based on NHANES data from 2007 to 2012 [16]. Similarly, few other US studies have investigated the prevalence of nonvaccine-type hrHPV infection following HPV vaccination programme among young women 13–26 years of age and none of them had found significant reduction of nonvaccine-type hrHPV infections [8, 17–19]. Of importance, all of these studies have not excluded the impact of previous sexual history on VE. In contrast, two other observational studies have suggested high bivalent VE against nonvaccine-type hrHPV 31/33/45 infections among women aged 20–22 years from Japan [20] and HPV31/35/45/52 among women aged 16–22 years from Netherlands [21]. In addition, the evidence for durability of cross-protection is limited to bivalent vaccine [22].

As the US has implemented the universal HPV vaccination programme for more than one decade, it is expected that such programme might show some VE against nonvaccine-type hrHPV infection. By using real-world data from NHANES from 2007 to 2016, this study aims to assess the effectiveness of quadrivalent HPV vaccine against genital vaccine-type HPV infection and its cross-protection against nonvaccine-type hrHPV infections among the noninstitutional women in US.

## Methods

### Data source

NHANES datasets from 2007 to 2016 were used for this study, including 5 surveys of 2-years [23]. The survey conducted by the Centers for Disease Control and Prevention (CDC), uses a multistage, stratified sampling design, combines data from interviews, physical examinations and laboratory tests to capture the health status of the US noninstitutionalised civilian population aged ≥2 years, with approximate 5000 persons participated each year. More details about NHANES survey could be found from the CDC website [23].

### Study population

For this study, female participants aged between 18 and 35 years were included in the analysis from the NHANES survey for years 2007–2016; 35 years was the oldest observed age for individuals who were vaccinated through 26 years of age (the oldest recommended age for vaccination in the United States) [6]. Participants were further restricted to those with available HPV vaccination history, laboratory testing for genital HPV and cervical cancer history from the NHANES. Vaccine history was obtained from the immunisation section of the questionnaire from the survey. Participants were excluded from the analysis if they did not know their vaccine history or refused to respond or did not have valid laboratory testing results for HPV or did not know or refused to respond cancer history.

### Variables and definitions

HPV vaccination history for participants was categorised into two groups (vaccinated for ≥1 dose of HPV vaccine and unvaccinated for no doses reported). The immunisation history from questionnaire data was also used to obtain age at receiving first vaccine dose, which was used to calculate time since vaccination (categorised into two groups: ≤4 years and between >4 and ≤10 years). Time since vaccination was calculated by subtracting age at interview from age at receipt of first vaccine dose. As first HPV vaccine was only available since year 2006, the calculated duration was considered valid only if it was ≤10 years otherwise categorised as unknown. In addition, although the survey did not have age at receiving first vaccine dose for participants in 2007–2010, vaccinated participants in this period would not be possible to have received their vaccine more than 4 years and therefore they were categorised into ≤4 years group for the analysis of time since vaccination.

Sexual behaviour from questionnaire data was also used for this study. Vaccinated participants were categorised by sexual debut according to age at receipt of first vaccine dose and age at having first sexual intercourse. Participants were considered as vaccinated before sexual debut if age at receiving first vaccine dose was equal or younger than age at initiating sexual intercourse, otherwise considered as vaccinated after sexual debut. For vaccinated participants who have not had any sexual intercourse at interview, they were considered as vaccinated prior to sexual debut. However, participants aged 18–19 years in NHANES 2007–2008 were not available for their sexual behaviour from the public available data. Therefore, all participants in this age range from 2007–2008 were categorised as unknown for the analysis by sexual debut as well as other variables from sexual behaviour questionnaire for this age group.

Medical conditions from questionnaire data were used to identify cervical cancer. Participants were considered as having cervical cancer if ever being told by doctors or other health professionals had cervical cancer. The age of cervical cancer diagnosis was also recorded.

Laboratory data were used to ascertain testing results by using self-collected cervicovaginal sample. Extracted DNA was evaluated for the presence of 37 HPV genotypes using PGMY09/11 polymerase chain reaction and Roche Linear Array [24]. All samples were hybridised to the typing strip for qualitative detection of 37 HPV types (6/11/16/18/26/31/33/35/39/40/42/45/51/52/53/54/55/56/58/59/61/62/81/82/83/84/89/IS39) and beta-globin (control for sample amplification). Samples tested negative for both HPV and beta-globin were considered inadequate. Given the predominant use of the quadrivalent vaccine in US through 2016 [7], test results were used to define vaccine-type HPV16/18/6/11 infection and nonvaccine-type hrHPV infection with any of the following 13 hrHPV types, including 31/33/35/39/45/51/52/56/58/59/68/73/82 according to their oncogenic potential [2]. For this study, demographics data was used to obtain the following basic socio-demographic information of the participants, age at diagnosis (18–22, 23–27, 28–35 years), race/ethnicity (non-Hispanic white, Hispanic, non-Hispanic black and other race), country of birth (born in US, born outside the US), poverty level (<1.0 below the national poverty level, between ≥1.0 and <5, ≥5.0), marital status (never married, live with partner and married, divorced, widowed or separated), education (< high school, high school or general equivalency diploma, > high school). Questionnaire data was used to obtain health insurance (yes or no), health condition (poor, fair, good, very good and excellent), health care utilisation (number of physician visits during the previous 12 months, 0, 1–3, 4–9, ≥10 visits), total number of lifetime sexual partners (≤2, ≥3) and age at sexual debut (<15, 15–17, >17 years).

### Statistical analysis

All analyses were conducted with weighted data as previously [25]. Weights were given by NHANES for each 2-year cycle and combined to create 10-year weights to be applied at appropriate steps in analysis as recommended by NHANES statistical guidelines [26].

The prevalence of HPV vaccine, vaccine-type HPV16/18/6/11 and nonvaccine-type hrHPV (HPV31/33/35/39/45/51/52/56/58/59/68/73/82) infections were calculated from 2007 to 2016 for all participants and by sociodemographic information, health condition and healthcare seeking behaviour. Multivariate log-linear regression model and calculated PR were then used to assess the association of HPV vaccine with vaccine-type HPV infections and nonvaccine-type hrHPV infections by adjusting the potential characteristic variables. Characteristics with a univariate *P* < 0.1, known associations with HPV infection (age, race/ethnicity, marital status, total number of lifetime sexual partners, age at sexual debut), or of interest for this study (HPV vaccination and survey cycle) were evaluated in multivariate modelling. Backward elimination (*P* < 0.05) was used to select the adjusted multivariate models, retaining covariates related to HPV infection or variables of interest to this study, regardless of statistical significance.

VE against HPV infection was calculated as (1-adjusted PR) × 100% as previously [27, 28]. We estimated VE against vaccine-type HPV infection overall, by age at diagnosis (18–22, 23–27 and 28–35 years), time since vaccination (≤4, between >4 and ≤10 years compared with unvaccinated), sexual debut (vaccinated after sexual debut, vaccinated before sexual debut compared with unvaccinated) and lifetime sexual partners (≤2, ≥3).

For the analysis of cross-protection against nonvaccine-type hrHPV infection, the study population was restricted to participants aged 18–22 years who have not received any HPV vaccine or had received their first vaccine dose prior to sexual debut. The VE against all 13 nonvaccine type hrHPV infection and selected nonvaccine-type hrHPV was investigated by time since vaccination and lifetime sexual partners.

All the analysis were conducted in R (version 4.1.3) using the survey package. A two-sided *P* value of <0.05 was considered statistically significant.

### Ethical statement

Because this study used deidentified, publicly available data, it did not meet the definition of human subjects research of the institutional review board, and therefore ethical review and informed consent were not required.

## Results

**Fig. 1.**
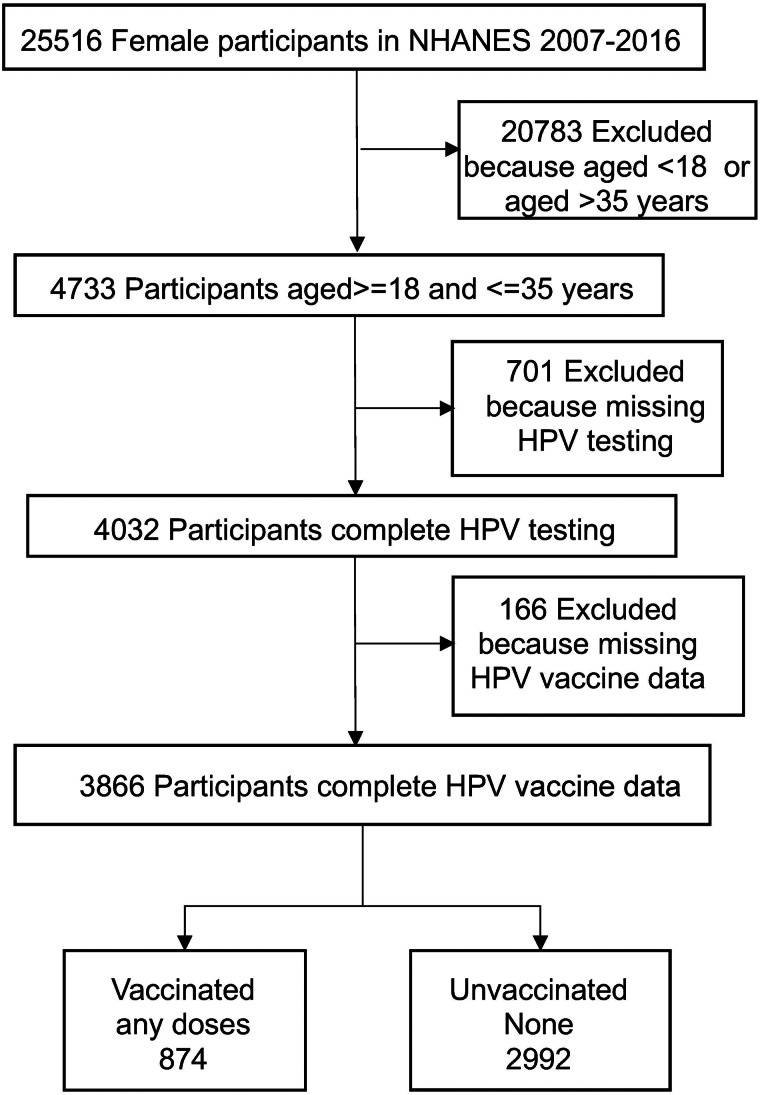
Flow chart of participants in National Health and Nutrition Examination Survey (NHANES) from 2007 to 2016. HPV, human papillomavirus.

From 2007 to 2016, a total of 25 516 female participants were enrolled in NHANES, of whom 3866 aged 18–35 years completed HPV testing and survey questionnaires for HPV vaccination were included in this study (Fig. 1).

Table 1 shows the participants who were vaccinated or had vaccine-type HPV (HPV18/16/11/6) infection or nonvaccine-type hrHPV (HPV31/33/35/39/45/51/52/56/58/59/68/73/82) infections according to various characteristics. A total of 874 (23.3%, 95% CI 21.3%–25.4%) were vaccinated with at least one dose in the study period (Table 1). Participants were more likely to be vaccinated with the following characteristics, including being younger, of non-Hispanic white, being born in the US, having higher education level (>high school), having a higher poverty index (≥5), having health insurance, better health condition, more health visits in the last year, never married or living with partner and having first sexual intercourse aged less than 15 years (Table 1 and Supplementary Table S1). Over the study period, the overall prevalence of vaccine-type HPV infection was 9.3% (95% CI 8.3%–10.4%) and the prevalence of nonvaccine-type hrHPV infection was 25.4% (95% CI 23.6%–27.3%) (Table 1).

**Table 1.** Prevalence of human papillomavirus vaccination, 4 valent vaccine-type HPV infection and cervical cancer overall and by characteristics, US, 2007–2016

	Sample size	No. vaccinated	Prevalence, % (95% CI)	No. vaccine-type HPV	Prevalence, % (95% CI)	No. nonvaccine-type hrHPV	Prevalence, % (95% CI)
Overall	3866	874	23.3 (21.3–25.4)	369	9.3 (8.3–10.4)	1025	25.4 (23.6–27.3)
Age							
18–22	1208	466	39.9 (35.9–44)	132	11.1 (9–13.4)	387	31.1 (27.9–34.4)
23–27	1008	256	27.4 (23.8–31.2)	92	9.5 (7.5–11.9)	284	28.5 (24.9–32.4)
28–35	1650	152	10.4 (8.6–12.5)	145	8.1 (6.8–9.6)	354	20 (17.9–22.1)
Age at first intercourse, y							
<15	999	263	25.8 (22.7–29.1)	112	10.4 (8.4–12.7)	309	29.8 (26.5–33.3)
15–17	1073	254	24.8 (21.4–28.5)	111	11.1 (9–13.6)	336	29 (25.5–32.6)
>17	1057	201	19.6 (16.4–23)	79	7.8 (5.9–10)	243	21.7 (18.6–25.1)
Unknown	737	156	23.1 (19–27.6)	67	7.1 (5.2–9.4)	137	19 (15.6–22.7)
Number of lifetime sex partners							
≤2	1161	297	25 (21.5–28.6)	38	2.6 (1.8–3.5)	147	10 (8.2–12)
≥3	2158	495	23.7 (21.2–26.3)	265	12.4 (10.9–13.9)	742	32.5 (30.1–34.9)
Unknown	547	82	17.3 (13.1–22.1)	66	9.5 (7.1–12.4)	136	25.5 (21.2–30.2)
Years of survey							
2007–2010	1566	191	12.6 (10.3–15.1)	212	13.1 (11.5–14.8)	443	27.9 (24.8–31.1)
2011–2014	1551	424	27.1 (23.4–31)	123	7.7 (6.1–9.4)	424	25.9 (23.1–28.9)
2015–2016	749	259	36 (31.2–41)	34	5.5 (3.9–7.6)	158	19.9 (16.4–23.8)

*Vaccine-type HPV included types 18/16/11/6, nonvaccine-type high-risk HPV (hrHPV) included HPV31/33/35/39/45/51/52/56/58/59/68/73/82.

**Fig. 2.**
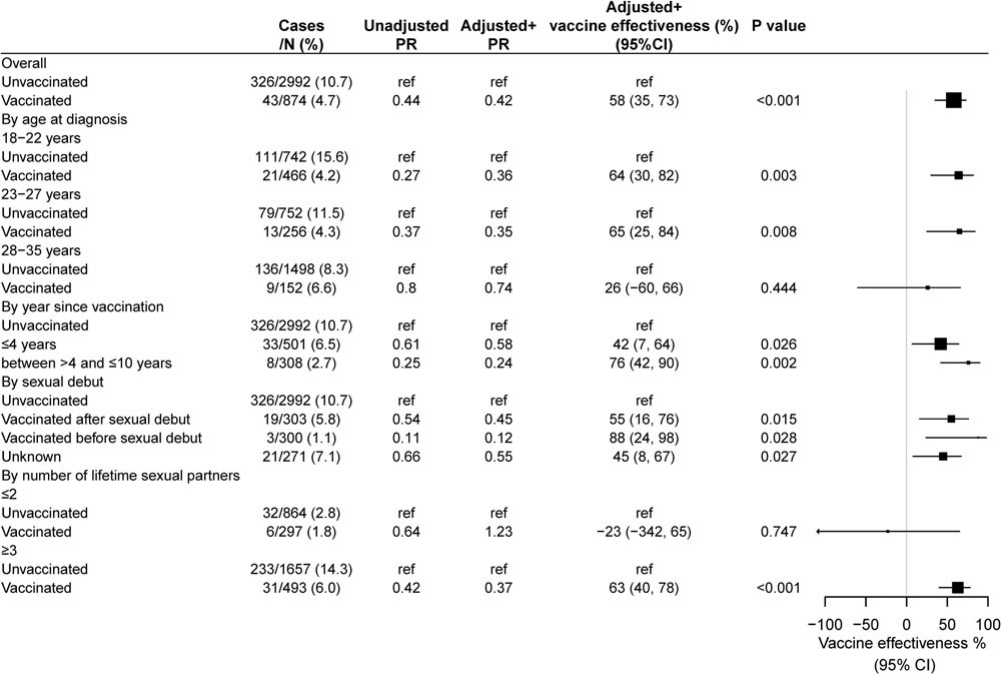
Vaccine effectiveness against vaccine-type human papillomavirus (HPV) infection. Vaccine-type HPV infection included: 18/16/11/6. ^+^Adjusted for age, marital status, insurance, number of total sex partner, year of survey. HPV, human papillomavirus; PR, prevalence ratio; CI, confidence interval; ref, reference.

The overall VE against vaccine-type HPV infection was 58% in the adjusted analysis. When the analysis was stratified by age at diagnosis of HPV, VE was 64% (95% CI 30%–82%, *P* = 0.001) in those aged 18–22 years and 65% (95%CI 25%–84%, *P* = 0.015) among those aged 23–27 years, while no effect was found among those aged 28–35 years. In the analysis according to time since vaccination, the VE increased with year since vaccination from 42% among those vaccinated within 4 years to 76% among those vaccinated 5–10 years ago. As for the analysis by vaccination in relation to sexual debut, the VE was 88% among participants received HPV vaccine prior to sexual debut and 55% among participants received vaccine after sexual debut. While VE was not found for those with ≤2 lifetime sexual partner, it was 63% for participants with ≥3 sexual partners (Fig. 2).

For the analysis of cross-protection against nonvaccine-type hrHPV infection, a total of 3292 participants aged 18–35 years were included in the analysis with 2992 unvaccinated and 300 vaccinated prior to sexual debut. No significant association was found in the overall analysis. However, the VE was 47% (95% CI 23%–64%) in participants aged 18–22 years and no effect was found in other age groups (Table 2).

**Table 2.** Vaccine effectiveness against nonvaccine-type high risk HPV (hrHPV) infection. Nonvaccine-type hrHPV infection included HPV31/33/35/39/45/51/52/56/58/59/68/73/82

	*n*/*N* (%)	Un adjusted PR	Adjusted PR (95% CI)	Adjusted vaccine effectiveness % (95% CI)
Overall				
Unvaccinated	738/2992 (23.1)	ref	ref	
Vaccinated	51/300 (16.7)	0.72	0.81 (0.58–1.12)	19 (−12 to 42)
By age				
18–22 years				
Unvaccinated	236/742 (31.3)	ref	ref	
Vaccinated	36/225 (13.8)	0.44	0.53 (0.36–0.77)	47 (23–64)
23–27 years				
Unvaccinated	196/752 (24.8)	ref	ref	
Vaccinated	11/66 (22.1)	0.89	1.65 (0.95–2.88)	−65 (−188 to 5)
28–35 years				
Unvaccinated	306/1498 (18.9)	ref	ref	
Vaccinated	4/9 (35.7)	1.89	1.97 (0.80–4.88)	−97 (−388 to 20)
By year since vaccination				
Unvaccinated	738/2992 (23.1)	ref	ref	
≤4 years	16/131 (10.4)	0.45	0.32 (0.1–0.97)	68 (3–90)
between >4 and ≤10 years	35/165 (20.4)	0.88	0.87 (0.55–1.38)	13 (−38 to 45)
By number of lifetime sexual partners				
≤2				
Unvaccinated	103/864 (9.4)	ref	ref	
Vaccinated	22/201 (8.8)	0.94	0.77 (0.34–1.76)	23 (−76 to 66)
≥3				
Unvaccinated	526/1657 (29.3)	ref	ref	
Vaccinated	29/99 (27.9)	0.95	0.76 (0.51–1.15)	24 (−15 to 49)

Adjusted for age, insurance, number of lifetime sex partner and year of survey. PR, prevalence ratio.

**Fig. 3.**
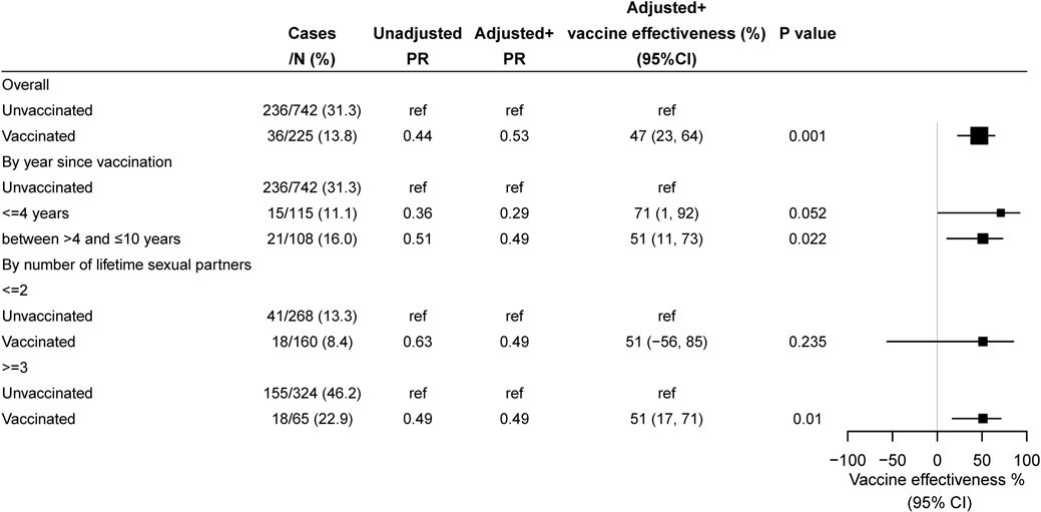
Vaccine effectiveness against nonvaccine-type high-risk human papillomavirus (hrHPV) infection among participants aged 18–22 years. Nonvaccine-type hrHPV infection included HPV31/33/35/39/45/51/52/56/58/59/68/73/82. ^+^Adjusted for age, insurance, number of total sex partner, year of survey. HPV, human papillomavirus; PR, prevalence ratio; CI, confidence interval; ref, reference.

We therefore further restricted our sample to participants aged 18–22 years for the subgroup analysis of cross-protection, including 742 unvaccinated and 225 vaccinated prior to sexual debut (Fig. 3). The VE against nonvaccine-type hrHPV infection was found at 47% (95% CI 23%–64%). In the subgroup analysis by time since vaccination, VE was 71% (95% CI 1%–92%) for those vaccinated within 4 years and 51% (95% CI 11%–73%) for those vaccinated 5–10 years.

**Fig. 4.**
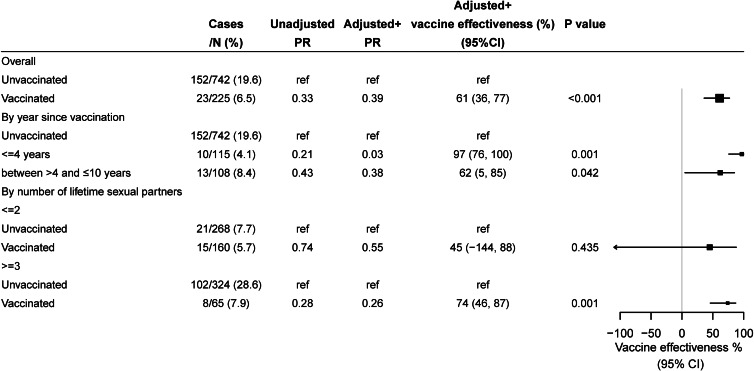
Vaccine effectiveness against 5 selected HPV35/39/52/58/59 infection among participants aged 18–22 years. ^+^Adjusted for age, insurance, number of total sex partner, year of survey. HPV, human papillomavirus; PR, prevalence ratio; CI, confidence interval; ref, reference.

We also evaluated the type-specific VE for the individual nonvaccine-type hrHPV infection (Table 3). Some individual effectiveness was found at 88% (95% CI 45%–97%) for HPV35, 66% (95% CI 8%–87%) for HPV39, 56% (95% CI −5% to 82%) for HPV52, 64% (95% CI −14% to 89%) for HPV58, 70% (95% CI 39%–85%) for HPV59 although without statistical significance due to small sample size for HPV52 and HPV58. The crude VEs against 5 selected nonvaccine-type hrHPV 35/39/52/58/59 were statistically significant at 67% (95% CI 47%–79%). The adjusted VE against nonvaccine-type hrHPV 35/39/52/58/59 was at 61% (95% CI 36%–77%) (Fig. 4). In the subgroup analysis by time since vaccination, VE was 97% (95% CI 76%–100%) for those vaccinated within 4 years and 62% (95% CI 5%–85%) for those vaccinated 5–10 years.

**Table 3.** Vaccine effectiveness (VE) against individual high-risk human papillomavirus (hrHPV) infection among participants aged 18–22 years

Variable	Vaccinated, No. (%) (*n* = 225)	Unvaccinated, No. (%) (*n* = 742)	PR (95% CI)	Vaccine effectiveness, % (95% CI)	*P* value
HPV16	1 (0.3)	67 (11.1)	0.03 (0–0.19)	97 (81–100)	0.001
HPV18	1 (0.2)	17 (2.0)	0.11 (0.01–0.86)	89 (14–99)	0.039
HPV31	4 (1.4)	25 (2.8)	0.49 (0.16–1.48)	51 (−48 to 84)	0.211
HPV33	1 (0.8)	6 (1.0)	0.87 (0.09–7.98)	13 (−698 to 91)	0.901
HPV35	2 (0.6)	29 (4.6)	0.12 (0.03–0.55)	88 (45–97)	0.008
HPV39	7 (2.5)	46 (7.2)	0.34 (0.13–0.92)	66 (8–87)	0.038
HPV45	3 (2.4)	13 (1.7)	1.4 (0.35–5.61)	−40 (−461 to 65)	0.636
HPV51	11 (4.7)	52 (7.5)	0.63 (0.27–1.46)	37 (−46 to 73)	0.284
HPV52	7 (2.0)	44 (4.6)	0.44 (0.18–1.05)	56 (−5 to 82)	0.068
HPV56	2 (1.2)	36 (3.9)	0.31 (0.05–1.83)	69 (−83 to 95)	0.200
HPV58	3 (0.9)	21 (2.7)	0.36 (0.11–1.14)	64 (−14 to 89)	0.086
HPV59	7 (1.4)	44 (4.7)	0.3 (0.15–0.61)	70 (39–85)	0.001
HPV68	4 (1.0)	22 (2.6)	0.39 (0.12–1.24)	61 (−24 to 88)	0.115
HPV73	5 (3.3)	23 (3.2)	1.01 (0.29–3.46)	−1 (−246 to 71)	0.988
HPV82	4 (1.2)	12 (1.9)	0.62 (0.18–2.14)	38 (−114 to 82)	0.454

CI, confidence interval; PR, prevalence ratio.

This study has also tried to investigate the impact of HPV vaccination on cervical cancer from all included participants. We found the prevalence of cervical cancer ever diagnosed among vaccinated was significantly lower (0.46%, 4/874) than that among unvaccinated participants (1.27%, 38/2992). Of the 4 women with a history of cervical cancer diagnosis, 3 of them received HPV vaccine after the diagnosis of cervical cancer and the other one received HPV vaccine in the same year of diagnosis of cervical cancer.

## Discussion

In this study, we reported the overall VE against vaccine-type HPV infection was 58% among women aged 18 to 35 years from 2007 to 2016 based on the NHANES datasets, which was mainly found among participants aged 18–22 and 23–27 years. While cross-protection was not found against 13 nonvaccine-type hrHPV among all participants, substantial effectiveness was found among participants aged 18–22 years for the nonvaccine-type hrHPV infection overall and particularly for the following 5 individual types HPV 35/38/52/58/59. Both direct protection and cross-protection maintained effectiveness after 5–10 years of vaccination.

Consistent with previous studies [29], the quadrivalent HPV vaccine showed substantial effectiveness against vaccine-type HPV infection among participants aged 18–22 (VE = 64%) and 23–27 (65%) years while no effect was found among participants aged 28–35 years. The nonsignificant effect among participants aged 28–35 years were in line with previous reports among women in the age of 30–34 years based on NHANES data [9]. Such findings are expected as women in this age group were not vaccinated until at least at the age of 23 years through the catch-up vaccine programme, when most of them in this age group had initiated sexual activities (median 17 years, IQR 15–19 years) and 45% had at least 3 lifetime sexual partners [30], both of which increased the risk of HPV infection. In addition, high effectiveness was found among women vaccinated before sexual debut (VE = 88%). The high effectiveness among these participants highlighted the importance of initiating HPV vaccination before the exposure to HPV.

While a previous study reported that vaccinated young adult women (age 20–26 years) had a higher prevalence of nonvaccine-type hrHPV infection than unvaccinated women based on NHANES data, it did not exclude the impact of baseline HPV infection on this [16]. After restricting the analysis to sexually naive participants in our study, some cross-protection against nonvaccine-type hrHPV was found among participants aged 18–22 years. Such cross-protection was specially found against each of the following 5 nonvaccine-types of hrHPV 35/39/52/58/59. Therefore, it is important to exclude the risk of HPV infection at baseline in analysing cross-protection of HPV vaccine.

To our knowledge, cross-protection was mainly reported against HPV31/35/45/52 from bivalent HPV vaccine based on real-world data in Japan, Netherlands, Scotland and on clinical trial data in Costa Rica [20–22, 31]. Although most of these studies have also shown some weak protection against HPV39/58/59 in the range of 5% to 30% [17, 18], none statistically significant effect was found from these studies [22]. To date, one trial has examined the quadrivalent VE for cross protection and has only found some effect against HPV31 [29]. Therefore, our findings add to knowledge that quadrivalent vaccine could provide cross-protection against HPV35/39/52/58/59 based on real-world data in US noninstitutional population. The effect against these types is not surprising as HPV35/52/58 are genetically related to HPV16 and HPV39/59 are genetically related to HPV18. It has been suggested that anti-HPV16 and anti-HPV18 antibodies generated by vaccination might bind to and neutralise HPV virions genetically related to HPV16 and HPV18 [29].

In our study, the VE against nonvaccine-type hrHPV and vaccine-type HPV was similar or even higher among women who were vaccinated 5–10 years ago compared to women who were vaccinated within 4 years. These findings are consistent with those from Japan and Netherland, where high VE against the vaccine-types HPV16/18 and against HPV31/33/45 was observed up to 7 years after vaccination [20, 21]. Given the long duration of protection against both nonvaccine-type hrHPV and vaccine-type HPV infection, it is expected that HPV vaccine would have some impact on reducing cervical cancer and cervical intraepithelial neoplasia in the long term, which has been reported by some trials and observational studies [32–34]. Similarly, we found unvaccinated participants were about 2.8 times higher ever to have a diagnosis of cervical cancer than vaccinated participants (1.27% *vs* 0.46%). However, due to the limited number of participants with cervical cancer, we did not conduct some VE analysis in this study. We are expected to perform the analysis to confirm the effect on cervical cancer with larger sample size in the future rounds of survey.

While the HPV vaccination has shown strong protection against both vaccine-type HPV and nonvaccine-type hrHPV infection and some potential impact on reducing cervical cancer in US, globally only 15% of girls in the target age for HPV vaccination are fully protected in 2019 [35]. Out of the 87 countries with an available HPV vaccination estimate from WHO, only 16% and 12% of girls received the first and final dose of HPV vaccine from low- and middle-income countries comparing to 50% and 40% in high income countries [35]. And more evidence from these countries also suggested HPV vaccine is highly effective in preventing high-risk HPV infection from Kenya, Tanzania and India [36, 37]. To meet the target by 2030 for the global cervical cancer elimination strategy, it is therefore important to increase the introduction of HPV vaccination programme in low- and middle-income countries where access has been limited.

The strength of this study includes using large nationally representative samples from NHANES datasets with decreased selection bias. This provided comprehensive surveys and laboratory testing results in a consistent manner from 2007 to 2016. Several limitations are present in this study. One of the limitations is that only self-reported vaccination information is available from NHANSE. It is therefore recall bias could exist, which might have resulted in misclassification [38]. However, the vaccine uptake from these age groups is consistent with other reports based on recorded vaccination information [39] and this misclassification would be expected to be nondifferential by HPV infection status. Another limitation of this study is the lack of baseline HPV infection at the age of vaccine receipt. It is therefore the population for the analysis of cross-protection was restricted to participants who were vaccinated prior to sexual debut to decrease the impact of baseline infection. In addition, the sample size for type-specific positivity is quite small, which excluded us to investigate the duration of protection for individual type of HPV infection. However, the pooled analysis from this study suggested the quadrivalent HPV vaccine is effective against both vaccine-type and nonvaccine-type hrHPV infection for 5–10 years. Lastly, the NHANES did not has data about pap smear test or result, which excluded us to evaluate the impact of HPV vaccine on reducing cervical intraepithelial neoplasia and current cervical cancer. While the questionnaire from NHANES does allow us to investigate the prevalence of ever diagnosed cervical cancer by vaccination, the limited number of cervical cancers from the vaccinated women excluded us to conduct any subgroup analysis. Therefore, further study with larger sample size and longer period following vaccination is required to confirm these findings.

In conclusion, quadrivalent HPV vaccine does not only show substantial effectiveness against genital vaccine-type HPV among women aged 18–35 years, but also against nonvaccine-type hrHPV infections among those aged 18–22 years who were vaccinated before sexual debut. Such protection could last for 5–10 years following the receipt of HPV vaccine. These findings highlight the potential of significant reduction of cervical cancer following the universal HPV vaccination programme.

## Data Availability

All data used in this study is publicly available, which could be found from National Health and Nutrition Examination Survey (https://www.cdc.gov/nchs/nhanes/index.htm).

## Supplementary material

For supplementary material accompanying this paper visit http://doi.org/10.1017/S0950268823000213. click here to view supplementary material
