# Supplementary material for: Effectiveness of quadrivalent HPV vaccination in reducing vaccine-type and nonvaccine-type high risk HPV infection
Source: Epidemiol Infect. 2023 Feb 15;151:e37. doi: 10.1017/S0950268823000213 (PMC10028998; doi:10.1017/S0950268823000213)
Supplement: Supplementary file 1 [file S0950268823000213sup001.docx]

**Supplementary Table 1. Prevalence of human papillomavirus vaccination, 4 valent vaccine-type HPV infection, and cervical cancer overall and by characteristics, US, 2007-2016.**

|  | Sample size | No. vaccinated | Prevalence, % (95% CI) | No. vaccine-type HPV | Prevalence, % (95% CI) | No. nonvaccine-type hrHPV | Prevalence, % (95% CI) |
| --- | --- | --- | --- | --- | --- | --- | --- |
| Ethnicity | |  |  |  |  |  |  |
| Non-Hispanic White | 1356 | 326 | 25.5 (22.4, 28.8) | 136 | 9 (7.6, 10.5) | 330 | 23.9 (21, 26.8) |
| Hispanic | 882 | 211 | 22.1 (15.5, 21) | 115 | 13.3 (5.5, 9.4) | 330 | 36.6 (22.8, 28.3) |
| Non-Hispanic Black | 1157 | 218 | 18.2 (19.2, 25.3) | 85 | 7.3 (11, 15.9) | 290 | 25.5 (33.9, 39.4) |
| Other Race | 471 | 119 | 21.7 (17.4, 26.4) | 33 | 9.4 (6.1, 13.5) | 75 | 17.5 (13.2, 22.5) |
| Country of birth | |  |  |  |  |  |  |
| USA | 2964 | 749 | 25.2 (22.9, 27.5) | 315 | 9.9 (8.8, 11.1) | 840 | 26.2 (24.2, 28.2) |
| Overseas | 902 | 125 | 13.7 (10.7, 17.1) | 54 | 6.2 (4.4, 8.2) | 185 | 21.7 (18.7, 25) |
| Educational level | |  |  |  |  |  |  |
| <High school | 741 | 130 | 16.7 (13.2, 20.6) | 74 | 9.6 (7.1, 12.5) | 230 | 28.7 (25.1, 32.5) |
| High school or general equivalency diploma | 893 | 190 | 21.5 (17.8, 25.6) | 101 | 12.1 (9.5, 15.1) | 249 | 28.1 (24.7, 31.7) |
| >High school | 2226 | 553 | 25.3 (22.7, 28) | 191 | 8.3 (7, 9.7) | 544 | 23.8 (21.5, 26.3) |
| Poverty index | |  |  |  |  |  |  |
| <1 | 1169 | 250 | 21.8 (18.4, 25.4) | 136 | 11 (9.2, 13) | 353 | 29.1 (25.8, 32.5) |
| >1.0 and <5.0 | 1995 | 464 | 23.6 (21, 26.3) | 173 | 8.5 (7.1, 10.2) | 518 | 25.7 (23.2, 28.2) |
| ≥5 | 423 | 106 | 26.8 (21.6, 32.4) | 33 | 8.4 (5.6, 11.8) | 78 | 18.5 (13.7, 24.1) |
| Unknown | 279 | 54 | 16.9 (11.5, 23.5) | 27 | 12.7 (7.4, 19.6) | 76 | 27.7 (21.4, 34.6) |
| Health insurance | |  |  |  |  |  |  |
| No | 1097 | 158 | 15.5 (12.8, 18.4) | 137 | 13.6 (10.9, 16.7) | 323 | 30 (26.5, 33.7) |
| Yes | 2759 | 713 | 25.8 (23.5, 28.2) | 231 | 7.9 (6.8, 9.2) | 700 | 24 (21.7, 26.4) |
| Health condition | |  |  |  |  |  |  |
| Excellent | 604 | 152 | 23.6 (19.6, 28.1) | 46 | 8 (5.6, 11) | 151 | 24.8 (20.8, 29.2) |
| Very good | 1194 | 303 | 25.4 (22.2, 28.8) | 120 | 8.9 (7.3, 10.7) | 308 | 23.2 (20.1, 26.6) |
| Good | 1459 | 305 | 21.9 (19.3, 24.6) | 152 | 10.5 (8.7, 12.6) | 406 | 27.8 (25, 30.7) |
| Fair | 533 | 101 | 20.7 (16.7, 25.1) | 43 | 8.8 (5.9, 12.4) | 143 | 26.2 (21.8, 30.8) |
| Poor | 75 | 13 | 21.3 (9.9, 36.9) | 8 | 9.5 (3.4, 19.5) | 17 | 22.1 (11.9, 35.3) |
| Health visits in the last year | | |  |  |  |  |  |
| None | 552 | 72 | 14 (10, 18.6) | 61 | 10.4 (7.5, 13.9) | 134 | 23.1 (18.8, 27.7) |
| 1-3 | 1995 | 471 | 23.8 (21.3, 26.3) | 189 | 9.7 (8.3, 11.3) | 516 | 25.7 (23.3, 28.3) |
| 4-9 | 1097 | 269 | 25.8 (22.5, 29.3) | 103 | 8.3 (6.6, 10.3) | 329 | 27 (24, 30.3) |
| 10+ | 217 | 62 | 26.7 (19.8, 34.4) | 16 | 8.6 (4.6, 14.1) | 46 | 21.1 (15.4, 27.7) |
| Marital status | |  |  |  |  |  |  |
| Never married | 1356 | 360 | 29.4 (26, 33) | 169 | 13.7 (11.7, 15.9) | 468 | 34.8 (31.9, 37.8) |
| Live with partner | 518 | 107 | 24.2 (19.7, 29.2) | 56 | 10.2 (7.5, 13.4) | 150 | 30.6 (24.8, 36.9) |
| Married, widowed, divorced, or separated | 1411 | 154 | 12.4 (10.3, 14.7) | 98 | 5.9 (4.7, 7.2) | 240 | 15.3 (13.4, 17.3) |
| unknown | 581 | 253 | 46.2 (40.1, 52.4) | 46 | 6.5 (4.3, 9.3) | 167 | 26.5 (22, 31.3) |

^*^Vaccine-type HPV included types 18/16/11/6, nonvaccine-type high-risk HPV (hrHPV) included HPV31/33/35/39/45/51/52/56/58/59/68/73/82.
